# Oropharyngeal, proximal colonic, and vaginal microbiomes of healthy Korean native black pig gilts

**DOI:** 10.1186/s12866-022-02743-3

**Published:** 2023-01-05

**Authors:** Andrew Wange Bugenyi, Ma-Ro Lee, Yeon-Jae Choi, Ki-Duk Song, Hak-Kyo Lee, Young-Ok Son, Dong-Sun Lee, Sang-Chul Lee, Young-June Son, Jaeyoung Heo

**Affiliations:** 1 Department of Agricultural Convergence Technology, Jeonbuk National University, Jeonju, 54896 Republic of Korea; 2 National Agricultural Research Organization, Mbarara, Uganda; 3 Department of Animal Biotechnology, Jeonbuk National University, Jeonju, 54896 Republic of Korea; 4 International Agricultural Development and Cooperation Center, Jeonbuk National University, Jeonju, 54896 Republic of Korea; 5 The Animal Molecular Genetics and Breeding Center, Jeonbuk National University, Jeonju, 54896 Republic of Korea; 6 Department of Animal Biotechnology, Faculty of Biotechnology, College of Applied Life Sciences and Interdisciplinary Graduate Program in Advanced Convergence Technology and Science, Jeju National University, Jeju, 63243 Republic of Korea; 7 Jeju Microbiome Research Center, Jeju National University, Jeju Special Self-Governing Province, Jeju, 63243 Republic of Korea; 8 Faculty of Biotechnology, College of Applied Life Sciences and Interdisciplinary Graduate Program in Advanced Convergence Technology and Science, Jeju National University, Jeju, 63243 Republic of Korea; 9 Cronex Co, Cheongju, 28174 Republic of Korea

**Keywords:** Oropharyngeal, Vaginal, Proximal colon, Microbiome, Jeju black pigs

## Abstract

**Background:**

Exploring the microbiome in multiple body sites of a livestock species informs approaches to promote its health and performance through efficient and sustainable modulation of these microbial ecosystems. Here, we employed 16S rRNA gene sequencing to describe the microbiome in the oropharyngeal cavity, proximal colon, and vaginal tract of Jeju Black pigs (JBP), which are native to the Korean peninsula.

**Results:**

We sampled nine 7-month-old JBP gilts raised under controlled conditions. The most abundant phyla that we found within the oropharyngeal microbiota were *Proteobacteria*, *Bacteroidetes*, *Fusobacteria* and *Firmicutes,* collectively providing core features from twenty-five of their genera. We also found a proximal colonic microbial core composed of features from twenty of the genera of the two predominant phyla, *Firmicutes*, and *Bacteroidetes*. Remarkably, within the JBP vaginal microbiota, *Bacteroidetes* dominated at phylum level, contrary to previous reports regarding other pig breeds. Features of the JBP core vaginal microbiota, came from seventeen genera of the major phyla *Bacteroidetes*, *Firmicutes*, *Proteobacteria*, and *Fusobacteria.* Although these communities were distinct, we found some commonalities amongst them. Features from the genera *Streptococcus*, *Prevotella*, *Bacillus* and an unclassified genus of the family *Ruminococcaceae* were ubiquitous across the three body sites. Comparing oropharyngeal and proximal colonic communities, we found additional shared features from the genus *Anaerorhabdus*. Between oropharyngeal and vaginal ecosystems, we found other shared features from the genus *Campylobacter*, as well as unclassified genera from the families *Fusobacteriaceae* and *Flavobacteriaceae*. Proximal colonic and vaginal microbiota also shared features from the genera *Clostridium*, *Lactobacillus,* and an unclassified genus of *Clostridiales*.

**Conclusions:**

Our results delineate unique and ubiquitous features within and across the oropharyngeal, proximal colonic and vaginal microbial communities in this Korean native breed of pigs. These findings provide a reference for future microbiome-focused studies and suggest a potential for modulating these communities, utilizing ubiquitous features, to enhance health and performance of the JBP.

**Supplementary Information:**

The online version contains supplementary material available at 10.1186/s12866-022-02743-3.

## Background

The microbial ecosystems inhabiting sites on any multicellular organism, have an impact on the host’s physiology, immunity, and metabolism [1]. Composition of this microbiota is known to be influenced by factors such as the host’s species, breed, body site, age, diet, behavior, environment as well as management, in the case of livestock [2–4]. Research into this field has grown over the years due to advances in high-throughput sequencing and improvements in computational power [5]. At the core of contemporary research in this field, is an endeavor to explore how various factors mold these ecosystems and/or how the benefits of the microbiome to the host could be optimized [6, 7]. In livestock such as pigs, the goal is to promote health and performance through modulation of this microbiome. Majority of the interventions developed to modulate microbiota have mainly targeted the lower gastrointestinal tract (GIT). This is mostly due to the ease of application in this site. Oral administration of probiotics, prebiotics or synbiotics will directly influence composition within the GIT, especially the lower GIT where transit times are slower and allow for colonization. As we get further along, interest in, and attempts to modulate the microbiota in other body sites gains traction. Sites such as the oral pharyngeal cavity, upper respiratory tract, urinary and reproductive tracts among others are getting to the center of focus [8–12]. For example, some studies have provided evidence of the ability of probiotic bacteria to colonize other body sites such as the pharyngeal region [13, 14]. Henceforward, a thorough understanding of the hosts microbiome in several body sites is essential if we are to modify it efficiently and sustainably to the benefit of the host [15]. While the differences are expected, similarities in microbial patterns between these body sites remain poorly understood, yet modifications in one site have potential impacts in another or several other sites [16].

We bring attention to the Jeju Black pig because it is understudied compared to the commercial breeds of pigs that dominate the commercial piggery sector in the country. This native breed of pigs has been geographically isolated and conserved for generations on the Korean Island of Jeju [17]. Due to its genotypic uniqueness and adaptation [18–20], we believe approaches to modulate its microbiome can best be optimized by calibrating them using a breed-specific reference such as a description of its microbial characteristic.

The objective of this study was to define the microbiome of three body sites within the Jeju black pigs as a reference for future research in these animals, and development of approaches aimed at productivity enhancement through microbiome modulation. We use 16S ribosomal RNA gene sequencing techniques to study the microbiome in the oral cavity, the proximal colon, and the vaginal tract of 7-month-old gilts. With the 16S rRNA data, we further employed bioinformatic tools to predict functional composition of the microbiomes within these body sites.

## Results

### Sequence analysis

We analyzed the microbial DNA isolated from oropharyngeal cavities, proximal colons, and vaginal canals of nine 7-month-old Jeju Black Pig (JBP) gilts. A total of 3,024,215 sequences of the v3-4 hypervariable region of the 16S rRNA gene were obtained from all samples in this study. The output sequences ranged from 50,576 to 147,954 (mean 112,008 per sample). Following denoising, merging and removal of chimeric artifacts, we had a total of 1,327,844 high-quality sequences, with an average of 49,179.41 sequences per sample (12,411 to 70,828 sequences per sample). Of these, 456,326 belonged to samples taken from the oropharynx, 361,867 from the proximal colonic samples and 509,651 from the vaginal samples. A summary of quality filtering, merging and denoising statistics is presented in the Table S1.

### Diversity of the microbial communities in the three body sites

**Fig. 1:**
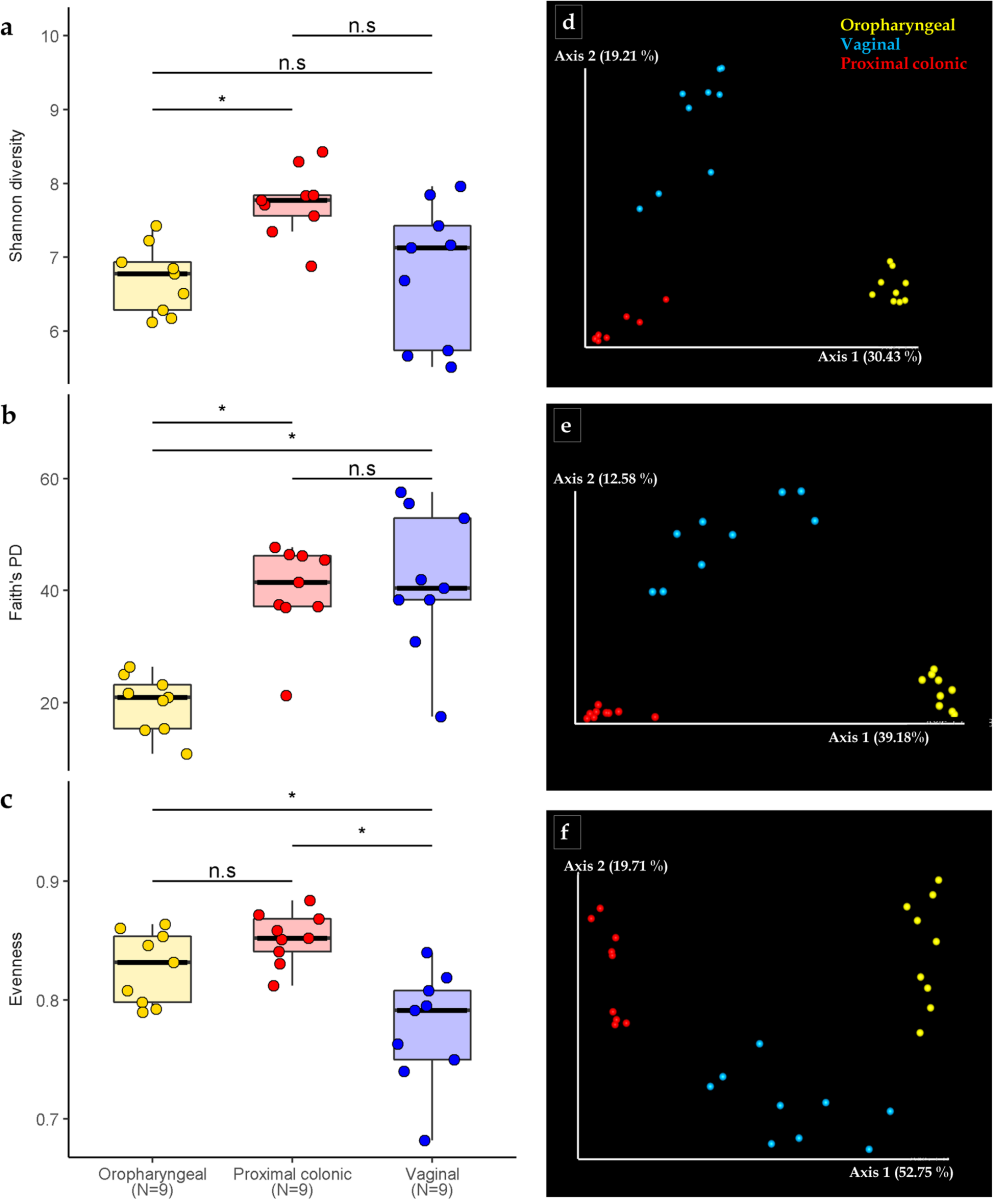
*Microbial diversity within the body sites*. Alpha diversity; (**a**) Shannon diversity, (**b**) Faith’s phylogenetic diversity and (**c**) Evenness within the samples. Beta diversity. Principal coordinate analysis (PCoA) based on (**d**) Bray–Curtis (**e**) unweighted UniFrac and (**f**) weighted UniFrac distances

Alpha and beta diversity indices are presented in Fig. 1. We compared alpha diversity between the groups using Shannon’s diversity, Faith’s phylogenetic diversity and Pielou’s evenness indices. Alpha diversity was lower in the oropharyngeal communities relative to both the proximal colonic and vaginal communities. This difference was significant according to Faith’s Phylogenetic diversity (*p*-value = 0.0015 when compared to proximal colon and *p*-value = 0.0019 when compared to vaginal communities) and only significant compared to proximal colonic samples according to Shannon’s index (*p*-value = 0.0015). Vaginal microbial communities had a significantly lower evenness compared to both the oropharyngeal (*p*-value = 0.04720) and proximal colonic (*p*-value = 0.00086) communities. Comparisons.

We also explored the distribution between samples using principal coordinate analysis plots based on Bray–Curtis distances as well as both unweighted and weighted UniFrac distance matrices (Fig. 1d- f). The samples tended to cluster by body site and the clustering appeared closer in both the proximal colonic and oropharyngeal samples relative to the vaginal communities. This effect was strongly revealed by the Bray–Curtis and unweighted UniFrac distances and less so when phylogenetic weights were applied. (Bray–Curtis pseudo-F = 10.64, *p*-value = 0.001; unweighted UniFrac pseudo-F = 11.48, *p*-value = 0.001; weighted UniFrac pseudo-F = 22.71, *p*-value = 0.001).

### Taxonomic composition of the three body sites

**Fig. 2:**
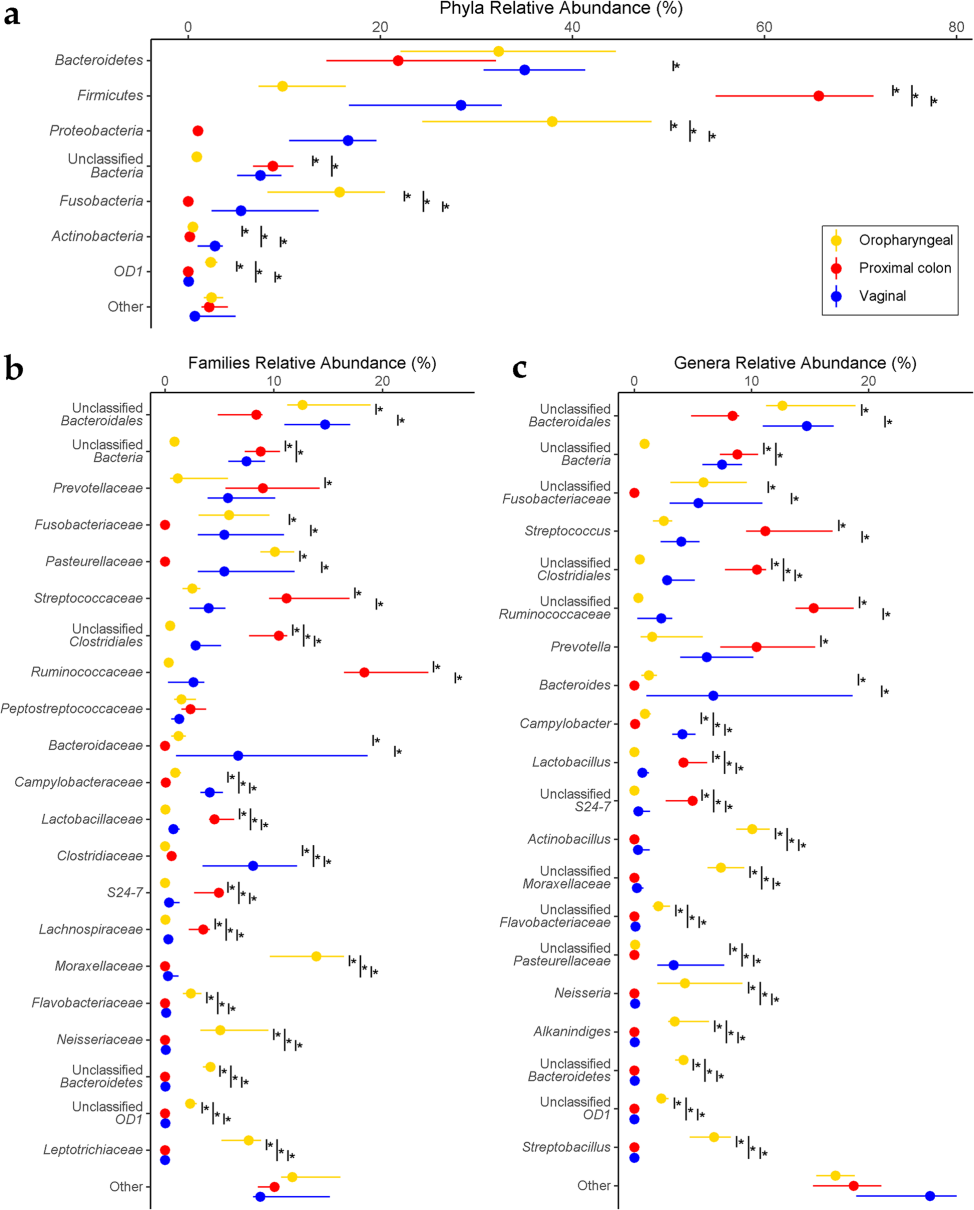
*Relative abundance of major taxonomic groups*: At (**a**) phylum, (**b**) family and (**c**) genus levels. At all these levels, taxa with maximum medians less than 2% across body sites were pooled into an “Other” category. The Asterix (*) indicates pairwise Wilcox test comparisons that were significant after correcting for multiple hypothesis testing (Benjamini–Hochberg method) at a cut-off of 0.05

The 1,327,844 quality filtered sequences were grouped into 4,215 amplicon sequence variants (ASVs). These ASVs/ features across all samples from all body sites then classified into 22 phyla, 111 families, and 206 genera. At the phylum level, 5 taxa with a relative abundance above 3% dominated the samples. These included, Firmicutes (33.2%), Bacteroidetes (30.6%), Proteobacteria 18.2%, Fusobacteria (7.4%), as well as an unclassified phylum of Bacteria (6.1%) The remaining 17 phyla made up only 4.5% of all features (Fig. 2a). At the genus level, 19 genera had mean relative abundances above 3% in at least one of the body sites. They included an unclassified *Bacteroidales* (13.34%), unclassified *Ruminococcaceae* (8.53%), *Streptococcus* (6.50%) unclassified *Bacteria* (6.13%) unclassified *Clostridiales* (4.67%) unclassified *Fusobacteriaceae* (4.55%), *Actinobacillus* (3.90%), *Prevotella* (3.74%), *Bacteroides* (3.47%), unclassified *Moraxellaceae* (3.15%), *Neisseria* (2.90%), *Streptobacillus* (2.69%), *Clostridium* (2.14%), *Lactobacillus* (2.03%), unclassified *Pasteurellaceae* (2.00%), unclassified *S24-7* (1.92%), *Campylobacter* (1.72%), *Alkanindiges* (1.67%) as well as an unclassified *Bacteroidetes* (1.44%) (Fig. 2c). To compare the distribution of the most abundant taxa across the body sites, we used their median since their distribution is non-normal. Figure 2 show the most abundant taxa with median distribution above 2% in at least one of the body sites. Of the 22 phyla found across all samples, a total of 17 were differentially distributed after correcting for multiple hypothesis testing. At lower taxonomic levels, 67 families and 115 genera were differentially distributed.

We used a negative binomial generalized linear model to test whether there were significant associations in distribution of genera within the body sites of the Jeju black pig gilts. To achieve this, we performed an exact test [21] in edgeR [22]. Our analysis revealed 84 genera whose patterns of distribution were significantly associated with the oropharyngeal communities relative to the other body sites. Twenty-five (25) of these had significantly higher relative abundances within the oropharyngeal cavity compared to their abundances in the other body sites. Among the proximal colonic communities, we detected 100 differentially distributed genera of which 15 had relative abundances that featured higher compared to other body sites. And finally in the vaginal communities, 47 genera showed differential patterns of distribution that were statistically significant. Forty-three (43) of these had higher relative abundances within the vaginal communities compared to the other body sites. The results of these tests are presented in supplementary tables (Tables S2-S4, FDR < 0.05, using the Bonferroni correction method).

### Core microbiota within these body sites

For this study’s purposes, we described core membership to a given body site as presence of a taxon in at least 8 out of the 9 samples (88.88% of the samples) collected from that site. Analysis at the amplicon sequence variants level revealed 82, 60, and 38 ASV’s core to the microbial ecosystems in the oropharyngeal cavity, proximal colon, and the vaginal canal respectively. These ASVs were annotated to 25, 20 and 17 genera within the oropharyngeal, proximal colonic and vaginal microbiota (Table 1). The core features within oropharyngeal microbial ecosystems belonged to 22 families from 7 phyla while those from the proximal colon belonged to 15 families from 4 phyla and those in the vaginal canal, belonged to 16 families from 5 phyla. For further insights into these communities, we probed for interesting taxa common to humans and/or other breeds of pigs as well as taxa associated with health and performance of pigs as reported in previous studies.

**Table 1:** Core genera defined as those present in at least 8 of the 9 samples (88.88% of the samples) from each body site

	**Core genera in the oropharyngeal cavity**	**Median Relative abundance (%)**		**Core genera in the proximal colon**	**Median Relative abundance (%)**		**Core genera in the vaginal canal**	**Median Relative abundance (%)**
1	Unclassified Bacteroidales	12.651	1	Unclassified Ruminococcaceae	15.331	1	Unclassified Bacteroidales	14.727
2	Actinobacillus	10.061	2	Streptococcus	11.179	2	Clostridium	8.211
3	Unclassified Moraxellaceae	7.383	3	Unclassified Clostridiales	10.462	3	Unclassified Bacteria	7.482
4	Streptobacillus	6.801	4	Prevotella	10.164	4	Bacteroides	6.725
5	Unclassified Fusobacteriaceae	5.886	5	Unclassified Bacteria	8.800	5	Prevotella	5.830
6	Neisseria	4.323	6	Unclassified Bacteroidales	8.380	6	Unclassified Fusobacteriaceae	5.458
7	Unclassified Bacteroidetes	4.187	7	Unclassified S24-7	4.956	7	Campylobacter	4.090
8	Alkanindiges	3.459	8	Lactobacillus	4.193	8	Streptococcus	3.992
9	Streptococcus	2.511	9	Clostridium	3.002	9	Unclassified Pasteurellaceae	3.365
10	Unclassified OD1	2.308	10	Ruminococcus	1.176	10	Unclassified Clostridiales	2.793
11	Unclassified Flavobacteriaceae	2.055	11	Bacillus	0.862	11	Unclassified Ruminococcaceae	2.299
12	Anaerorhabdus	1.713	12	Gemmiger	0.830	12	Peptostreptococcus	0.830
13	Unclassified Flavobacteriales	1.530	13	Faecalibacterium	0.591	13	Lactobacillus	0.688
14	Prevotella	1.515	14	Anaerorhabdus	0.514	14	Escherichia	0.605
15	Bacillus	1.410	15	Unclassified Erysipelotrichaceae	0.496	15	Bacillus	0.462
16	Unclassified SR1	1.350	16	Roseburia	0.427	16	Peptoniphilus	0.241
17	Bergeyella	1.328	17	Unclassified Lactobacillaceae	0.201	17	Unclassified Flavobacteriaceae	0.082
18	Unclassified Peptostreptococcaceae	1.032	18	Succinivibrio	0.166			
19	Campylobacter	0.899	19	Coprococcus	0.137			
20	Unclassified Bacteria	0.859	20	Propionispira	0.124			
21	Unclassified Alcaligenaceae	0.403						
22	Porphyromonas	0.384						
23	Unclassified Lactobacillales	0.355						
24	Unclassified Ruminococcaceae	0.328						
25	Unclassified Neisseriaceae	0.276						

### Ubiquitous genera across the body sites

**Fig. 3:**
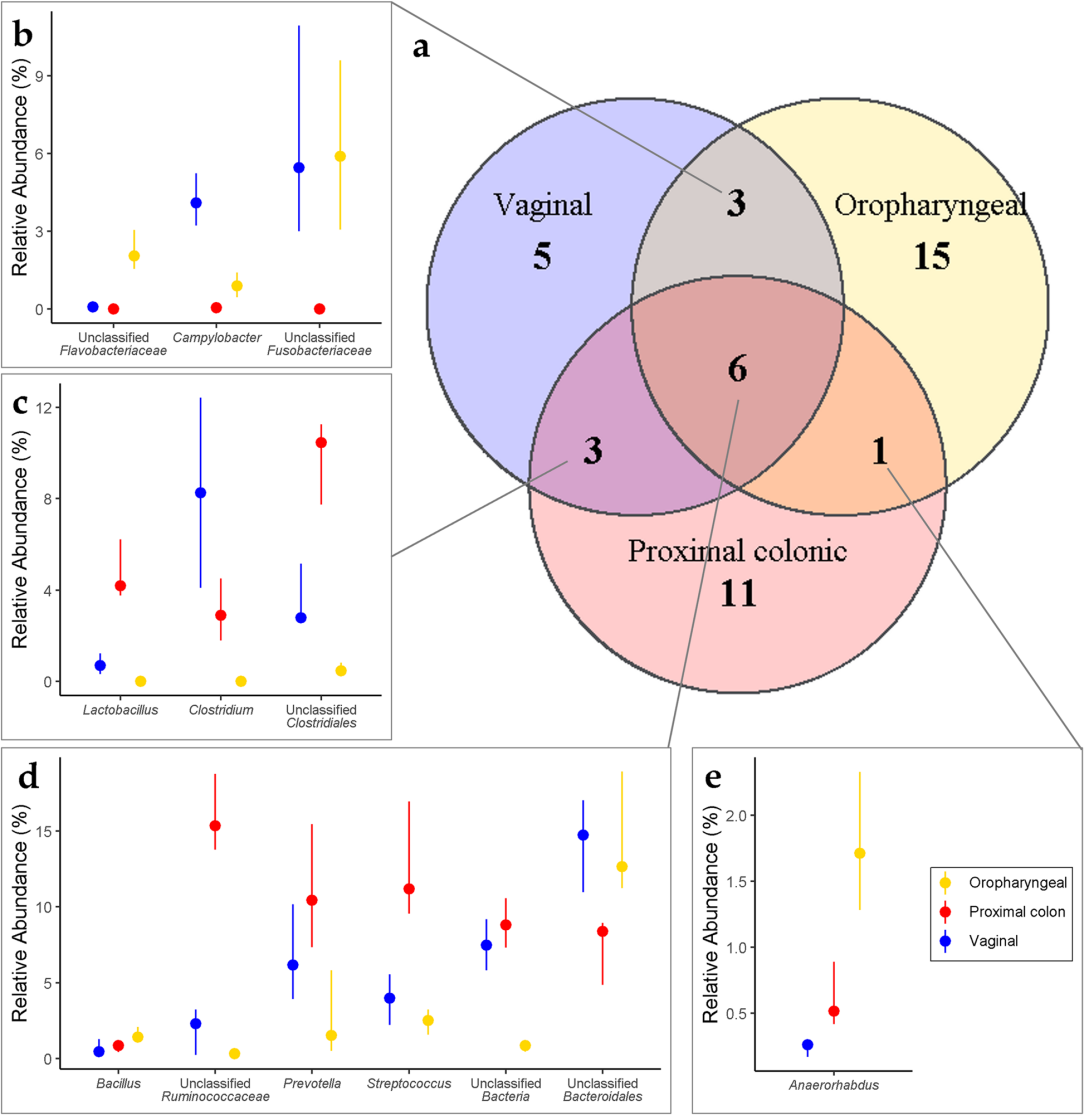
*Shared genera within the core microbiota in the three body sites.* The venn diagram (**a**) shows the number of genera within the core of the oropharyngeal, proximal colonic and vagina microbiota as well as those that are shared among the body sites. The point and range plots (**b**) shows the median and interquartile range of relative abundances of the genera within the core of the oropharyngeal and vaginal microbiota but not core in the proximal colon; (**c)**, core to the proximal colon and vaginal communities but not the oropharyngeal microbiota: (**d**) genera within the core of all three body sites; (**e)** core to the proximal colon and the oral communities but not the vaginal microbial communities

We also looked for genera that occupy several body sites in the Jeju black pigs (Fig. 3). Among the genera that were core to the studied body sites, we found only six (6) genera shared across all three studied body sites. These included *Streptococcus*, *Prevotella*, *Bacillus* an unclassified genus of the family *Ruminococcaceae*, an unclassified genus of the order *Bacteroidales* and an unclassified genus within the domain *Bacteria*, (Fig. 3d). These genera belonged to 3 phyla, *Bacteroidetes*, *Firmicutes*, and an unclassified Bacterial phylum (Table 2).

**Table 2:** Ubiquitous genera within the oropharyngeal cavity, proximal colon, and vaginal canal of 7-month-old Jeju Black Pig gilts

**Ubiquitous genera in the oropharyngeal, proximal colonic & vaginal microbial environments**						
		**Median relative abundances**				
	**Genus**	**oropharyngeal**	**Proximal colonic**	**Vaginal**	**Family**	**Phylum**
1	**Unclassified *Bacteroidales***	**12.651**	**8.38**	**14.727**	Unclassified *Bacteroidales*	*Bacteroidetes*
2	**Unclassified *Bacteria***	**0.859**	**8.8**	**7.482**	Unclassified *Bacteria*	Unclassified *Bacteria*
3	***Streptococcus***	**2.511**	**11.179**	**3.992**	*Streptococcaceae*	*Firmicutes*
4	***Prevotella***	**1.515**	**10.164**	**5.83**	*Prevotellaceae*	*Bacteroidetes*
5	**Unclassified *Ruminococcaceae***	**0.328**	**15.331**	**2.299**	*Ruminococcaceae*	*Firmicutes*
6	***Bacillus***	**1.41**	**0.862**	**0.462**	*Bacillaceae*	*Firmicutes*
**Ubiquitous genera in the oropharyngeal, and proximal colonic microbial environments**						
		**Median relative abundances**				
	**Genus**	**oropharyngeal**	**Proximal colonic**	**Vaginal**	**Family**	**Phylum**
1	***Anaerorhabdus***	**1.713**	**0.514**	0.260	*Erysipelotrichaceae*	*Firmicutes*
**Ubiquitous genera in the oropharyngeal, and vaginal microbial environments**						
		**Median relative abundances**				
	**Genus**	**oropharyngeal**	**Proximal colonic**	**Vaginal**	**Family**	**Phylum**
1	**Unclassified *Fusobacteriaceae***	**5.886**	0.000	**5.458**	*Fusobacteriaceae*	*Fusobacteria*
2	***Campylobacter***	**0.899**	0.054	**4.090**	*Campylobacteraceae*	*Proteobacteria*
3	**Unclassified *Flavobacteriaceae***	**2.055**	0.000	**0.082**	*Flavobacteriaceae*	*Bacteroidetes*
**Ubiquitous genera in the proximal colonic, and vaginal microbial environments**						
		**Median relative abundances**				
	**Genus**	**oropharyngeal**	**Proximal colonic**	**Vaginal**	**Family**	**Phylum**
1	**Unclassified *Clostridiales***	0.462	**10.462**	**2.793**	Unclassified *Clostridiales*	*Firmicutes*
2	***Clostridium***	0.000	**3.002**	**8.211**	*Clostridiaceae*	*Firmicutes*
3	***Lactobacillus***	0.009	**4.193**	**0.688**	*Lactobacillaceae*	*Firmicutes*

Only one genus, *Anaerorhabdus* from the family *Erysipelotrichaceae* and phylum, *Firmicutes* was core to both the oropharyngeal cavity and proximal colon but did not feature as core within the vaginal canal. Between the oropharyngeal cavity and the vaginal canal, we found 3 core genera that were not core to the proximal colonic microbiota. They included *Campylobacter*, as well as unclassified genera from the families *Fusobacteriaceae* and *Flavobacteriaceae*. These genera came from three separate phyla, *Proteobacteria*, *Fusobacteria* and *Bacteroidetes*. Comparing the core microbiota of the proximal colon and the vaginal canal showed an additional 3 genera shared between these communities but not core within the oropharyngeal environments. The genera, *Lactobacillus*, *Clostridium*, and an unclassified genus from the order, *Clostridiales* all belonged to the phylum Firmicutes (Fig. 3 and Table 2).

### Predicted microbial functional capacity

**Fig. 4:**
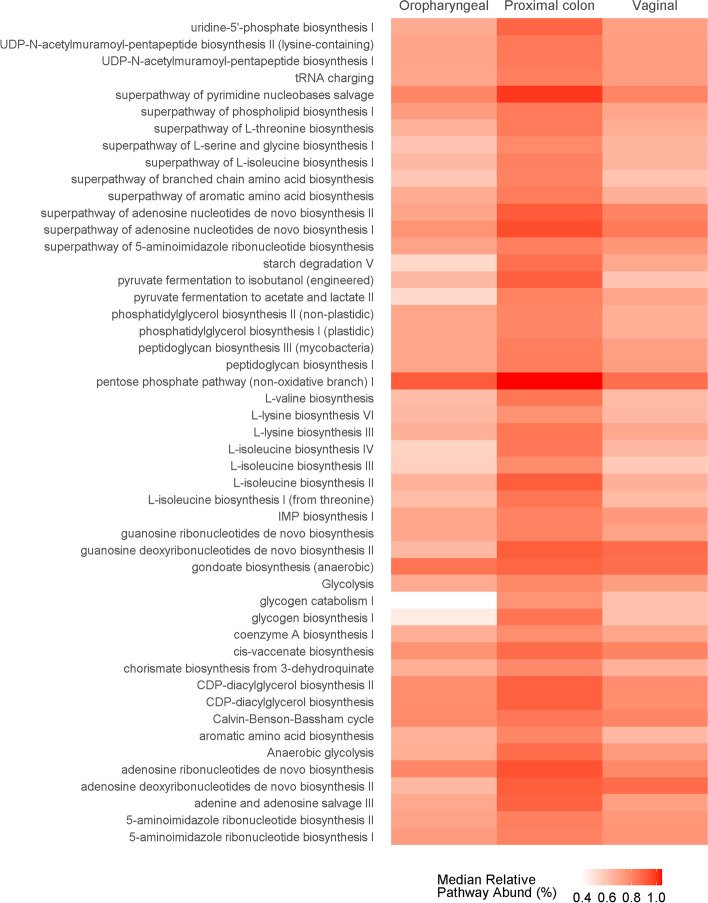
*Pathway abundances*. The heat map displays pathways whose median relative abundances reach at least 0.75% within one or more of the three body sites studied (oropharyngeal cavity, proximal colon, or vaginal tract) in the Jeju Black Pigs

**Fig. 5:**
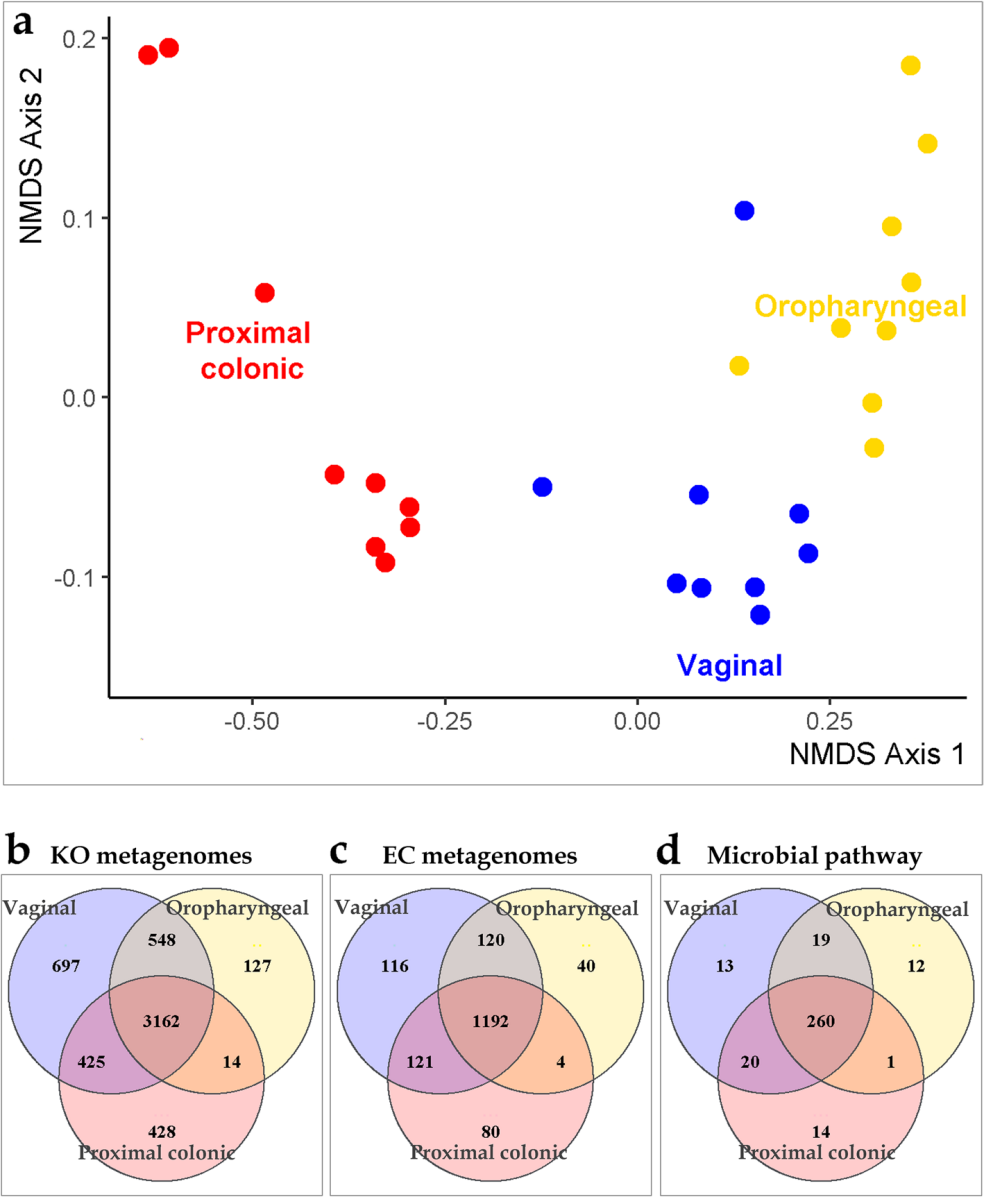
*Functionality analysis*: A non-metric dimensional scale (NMDS) plot (**a**) showing the distribution of samples based on Bray Curtis distances calculated using microbial pathway abundances. The venn diagrams show shared KEGG’s Orthologous metagenomes (**b**), Enzyme Committee (EC) metagenomes (**c**), and Pathway abundances (**d**) across the oropharyngeal, proximal colonic and vaginal microbial ecosystems

We used PICRUSt to predict functional composition of the microbial ecosystems in the Jeju black pigs body sites based on the 16S rRNA data. Our analysis yielded 392 predicted pathways from the 3 body sites. Of these 49 pathways had median relative abundances above 0.75% in at least one of the body sites (Fig. 4). The dominant pathways were annotated to 11 super pathway classes. These included Generation of precursor metabolites and energy, Amino acid biosynthesis, Fatty acid and lipid biosynthesis, Nucleoside and nucleotide biosynthesis, Cell structure biosynthesis, Aromatic compound biosynthesis, Polysaccharide degradation, Carbohydrate biosynthesis, Aminoacyl-tRNA charging, Carbohydrate degradation, as well as the Cofactor, carrier, and vitamin biosynthesis super pathways (Table S5). Consistent with taxonomic composition, diversity analysis of the microbial pathway abundances showed a clustering of samples that was strongly influenced by body sites (Fig. 5a Bray Curtis; PERMANOVA, pseudo-F = 26.5047, *p*-value = 0.001).

For insights into the differences across these body sites with respect to microbial functionality we fitted the MetaCyc predicted microbial pathway abundances to negative binomial generalized linear models. Analysis using an exact test in edgeR, revealed several pathways whose relative abundances were significantly associated with certain body sites (Tables S6-S8 FDR < 0.05, using the Bonferroni correction method). The analysis revealed 210 pathways from 31 super pathway classes with distribution patterns that were significantly associated with the microbiome in oropharyngeal cavities of the pigs. Among these, 77pathways from 24 super classes were more abundant within the oropharyngeal microbial ecosystems when compared to the proximal colonic and vaginal communities. Among the proximal colonic communities, 278 pathways from 32 super pathway classes showed significant differences in abundances relative to the microbiomes in the oropharyngeal and vaginal communities. A closer examination revealed that 120 of these pathways were associated with a relatively higher abundance within proximal colons of the pigs relative to the other two communities. The more abundant pathways belonged to 24 super pathway classes. A look at the vaginal microbiome showed 99 pathways from 22 super pathway classes that had relative abundance patterns that were significantly associated with the vaginal communities. Fifty-five (55) of these from 17 super pathway classes were relatively more abundant in this environment when compared to the other two body sites.

Using the same approach as that used with taxonomic data, we detected core microbial functional features across the three body sites. The core microbiome of the oropharyngeal, proximal colonic and vaginal communities consisted of 3,851, 4,029 and, 4,832, KO metagenomes respectively. We also detected 1,356, 1,397 and 1,549 core EC metagenomes as well 292, 295, 312 core MetaCyc pathways from the respective communities (Fig. 5b-d). Of these, 3,162 Ko metagenomes, 1,192 EC metagenomes and 260 MetaCyc pathways were shared across the three body sites (Fig. 5b-d). The 260 shared pathways belonged to thirty super pathways (Table S9). This shared group included interesting pathways such as those involved in alcohols degradation, antibiotic resistance, C1 compound utilization and assimilation, as well as inorganic nutrient metabolism among other pathways.

## Discussion

In this study, we present a comprehensive description of the microbial ecosystems in the oropharyngeal cavity, proximal colon, and vaginal canal of a relatively understudied native breed of pigs on the Korean island of Jeju. These body sites were selected because their microbial communities potentially influence function and health of the respiratory, digestive, and reproductive system in these animals [23]. Additionally, because of a putative cross talk that occurs between microbiota and their host, in ways that sometimes transcend body site [24, 25], we not only explored core membership of each community but also ubiquitous features across these three environments.

### Brief comparison of the body sites

The habitat provided within a body site is a strong determinant of microbial composition [26]. Despite the behavior of suids that includes interacting with groupmates and exploring their environments using their snout, diversity within oropharyngeal microbial communities was lower than that in the proximal colon and vagina. This implies that conditions within the oropharynx favor colonization by only a select group of microbes from the great range of environments that the animal gets exposed to. In the proximal colon, microbial communities tended to cluster closely and exhibited high diversities and evenness among them. On the other hand, although highly diverse, the vaginal communities had a low evenness and tended to cluster less closely relative to the oropharyngeal and proximal colonic samples. This variation within the vaginal samples is probably due to varying stages of the estrus cycle among the studied gilts. In the study design, we did not collect data on the estrus cycles of the gilts and there were no managemental attempts to synchronize estrus. We therefore assumed that the gilts were at various stages of their individual estrus cycles. Factors such as the increased immunoglobulin levels during the follicular phase of the cycle [27] are likely to influence bacterial attachment and overall vaginal microbial ecosystem [28]. In contrast to this hypothesis, Lorenzen and colleagues [29] found no variation in the vaginal microbiota in prepubertal and sexually mature minipigs. This contrast might be a peculiarity of the breed of pig studied in their experiment.

### Microbial composition in the oropharyngeal region

Within the oropharyngeal microbiota, *Proteobacteria*, *Bacteroidetes*, *Fusobacteria* and *Firmicutes* were the dominant phyla among our 7-month-old Jeju Black pig gilts making up 93.24% of the ASV’s. The phyla occupied 36.85%, 31.08%, 14.85%, and 10.46% respectively. The JBP gilts had a slight difference in oropharyngeal composition at the phylum level in comparison to findings from other breeds of domestic pigs (Table S11). In previous studies, *Firmicutes* occupied a more significant proportion of the oropharyngeal community, comparable only to *Proteobacteria* within these environments [30, 31]. The reason for this discrepancy could be attributed to the difference in breed, and age of the study animals since piglets and weaners were involved in the above-mentioned studies compared to the 7-month-old gilts.

Twenty-five genera were found in the oropharyngeal microbiota in at least 8 of the 9 pigs sampled and collectively made up a relative abundance of 75.01% within the oropharyngeal ecosystem. Most of the core features were contributed by families that were not only dominant within the oropharynx but also responsible for differences with the other body sites. For instance, *Moraxellaceae, Leptotrichiaceae, Neisseriaceae, Flavobacteriaceae* and an unclassified genus from the phylum *OD1* had significantly higher relative abundances in oropharyngeal samples than in any of the other two body sites. *Pasteurellaceae* and *Fusobacteriaceae*, were also significantly higher in the oropharyngeal than in the proximal colon although not significantly higher than in the vaginal communities. Interestingly, some of the core members were contributed by families occurring at relatively low abundances such as *Streptococcaceae, Prevotellaceae, Campylobacteraceae, Bacillaceae*, *Erysipelotrichaceae*, *Peptostreptococcaceae*, *Alcaligenaceae*, *Porphyromonadaceae*, *Ruminococcaceae* as well as an unclassified genus from the phylum, *SR1.*

For the core features that were classified at the genus level, we found overlaps with core genera of the swine tonsillar microbiome including genera such as *Actinobacillus, Alkanindiges, Streptococcus, Prevotella, Campylobacter* and *Porphyromonas* [32, 33]. Annotating the features to the family level, revealed stronger similarities to the core porcine tonsillar microbiome. Families such as *Pasteurellaceae*, *Moraxellaceae*, *Fusobacteriaceae*, *Neisseriaceae*, *Streptococcaceae*, *Peptostreptococcaceae*, *Prevotellaceae*, *Campylobacteraceae*, *and Porphyromonadaceae* were common to both our JBP oropharyngeal core and the swine tonsillar microbiota [32, 33]. The core community, however, did not include members of the families *Veillonellaceaea*, and *Treponemataceae as* reported in the core tonsillar community by the above studies. Our results are therefore an extension of this earlier work as our sample are taken from a broader community that encompasses and probably serves as a source of the tonsillar microbial community.

These core genera included some well-known members of the human oral microbiome such as *Prevotella*, *Streptococcus*, *Neisseria, Porphyromonas*, as well as members of the family, *Fusobacteriaceae*, and the phylum, *SR1* [34, 35]. Some of the genera *Streptococcus, Neisseria, Corynebacterium, Prevotella, Porphyromonas and Fusobacterium* have been linked with a ‘healthy oral microbiome’ in humans [36].

We looked for some commonly used probiotic genera such as *Bacillus, Lactobacillus, Bifidobacterium, Enterococcus, Pediococcus,* and *Streptococcus* [37]. We did find all these genera except *Bifidobacterium* within the oropharyngeal communities sampled*.* And of these, only *Bacillus,* and *Streptococcus* featured among the core community. This suggests an ability of these probiotic bacteria to attach and colonize in the oropharyngeal region.

### Microbial composition in the proximal colon

*Firmicutes*, and *Bacteroidetes* were the most abundant phyla in the proximal colons of the JBP gilts (~ 87% of all sequences). In this environment, the core features belonged to 20 genera including *Streptococcus*, *Prevotella*, *Lactobacillus*, *Clostridium*, *Ruminococcus*, *Bacillus*, *Gemmiger*, *Faecalibacterium*, *Anaerorhabdus*, *Roseburia*, *Succinivibrio*, *Coprococcus*, *Propionispira*. Also included were unclassified genera from the families *Ruminococcaceae*, *S24-7(Muribaculaceae)*, *Erysipelotrichaceae*, and *Lactobacillaceae* as well as unclassified genera from the orders *Clostridiales*, and *Bacteroidales*.

While several studies have described the core members of the intestinal tract [15, 38] (Table S12), our study is the first to focus on the proximal segment of the swine colon. Similar to these previous reports, we found core features from the genera *Prevotella*, *Lactobacillus*, *Clostridium*, *Ruminococcus*, and *Roseburia* [15, 38]. This therefore suggests that these genera are shared with the core of the entire swine gastro-intestinal tract. Our findings are therefore an extension of these studies and brings a higher resolution focus on the proximal colon.

### Microbial composition in the vaginal canal

*Bacteroidetes*, *Firmicutes*, *Proteobacteria,* and *Fusobacteria* were the most abundant phyla in the vaginal canal of the Jeju black pig gilts accounting for 86.75% of the sequences. A notable finding in our study was the dominance of *Bacteroidetes* over the *Firmicutes*. This was in contrast to findings from studies among other breeds of pigs [39–41] (Table S13). The glaring dominance of the *Bacteroidetes* were a peculiar feature in the JBP vaginal microbiota. Among other breeds of pigs, *Firmicutes* feature as the most dominant phylum followed by *Proteobacteria* and *Bacteroidetes* especially among sows [40, 41]. In their study, Wang *et. al* [40]*,* link the proliferation of *Bacteroidetes* and *Proteobacteria* to endometritis among postpartum sows. The difference between the vaginal microbiota in our studied gilts and previous reports is likely due to difference in age group studied [42] as well as due to breed differences. The vaginal microbiota is known to be markedly influenced by the reproductive phase of a pig given that hormonal levels and reproductive tract secretions significantly alter the vaginal environment [28].

The core features within the vaginal microbial environment of our JBP gilts were annotated to the genera *Clostridium*, *Bacteroides*, *Prevotella*, *Campylobacter*, *Streptococcus*, *Peptostreptococcus*, *Lactobacillus*, *Escherichia*, *Bacillus*, *Peptoniphilus*. Others belonged to unclassified genera from the families *Fusobacteriaceae*, *Pasteurellaceae*, *Ruminococcaceae*, and *Flavobacteriaceae* as well as genera from the orders *Bacteroidales*, and *Clostridiales*. Despite differences at phylum level, the vaginal microbiome of our JBP had similarities with that of commercial pig breeds at the genus level. Some of the core features in this community belonged to *Clostridium, Streptococcus, Lactobacillus, Bacillus,* and an unclassified genus of *Fusobacteriaceae* which show up as core members of the vaginal microbiota in gilts of commercial breeds [42]. As with other breeds of domestic pigs and other non-human mammals the abundance of Lactobacillus was relatively low within the vaginal microbiota of the JBP gilts compared to that in humans [43].

We were also interested in identifying features with a possible influence on reproductive performance within the core of our JBP gilt vaginal microbiome. Our data revealed some taxa, such as *Bacteroides*, *Prevotella*, *Campylobacter*, and *Ruminococcaceae* that had been identified as potential biomarkers of performance based on certain reproductive parameters [44].

### Ubiquitous features within the body sites

Microbial features belonging to 6 genera occupied all the three body sites. This was interesting considering the variability in physiology, function and biochemical characteristics of the oropharyngeal cavity, proximal colon, and vaginal canal. The features were annotated to *Firmicutes* and *Bacteroidetes* phyla which dominated all the three body sites among these JBP gilts. The ubiquitous nature of these microbial features suggests an ability to colonize multiple body sites.

Several factors enable intermingling and transfer of microbiota across body sites and between individuals in domestic pigs. One is the social interaction among grouped pigs which is a common rearing practice at this stage of the pig’s life. The high degree of interaction increases the transferability of microbiota between these three body sites as pigs groom each other and interact with manure-smeared floors, surfaces, and various objects within their environment [4]. In such interactions, microbial organisms from the gut could ascend from the rectum into the vaginal canal, and grooming groupmates could lead to ingestion of gut microbes from fecal matter, vagina/ vulva as well skin and other parts of the pig’s body. Moreover, a key managemental practice among this age group is acclimatization of replacement gilts on introduction into a breeding farm. This involves, among many practices, exposure of replacement gilts to fresh fecal and placental material from older breeding sows as a means of inducing immunity against endemic pathogens on the receiving farm [45, 46].

Ubiquitous members of these ecosystems are also important to understand since they are likely to enable a vertical transfer of microbiota to newborn piglets. Following recruitment of gilts into the breeding herd, their vaginal and fecal microbiota will be the first encountered by neonate piglets and is believed to seed the microbiota of the new pigs. Evidence supporting this is found in the fact that the pharyngeal microbiota of neonates tends to look more like that within the vaginal tract of their dams [47, 48]. These phenomena among others, make it interesting to understand the commonality that might exist within these body sites, among gilts at a period when they enter the reproductive cycle.

### Predicted functionality

In addition to knowledge on phylogenetic composition, it is important to understand the potential contribution that a microbial community brings to a host’s physiology and health. As expected, our analysis showed that pathway abundances generally clustered by body sites, corroborating the strong effect of body site on the microbiome of Jeju black pigs. Our exploration of the microbial pathways revealed several common metabolic reactions (260 pathways in 30 super pathways) in these 3 body sites. These ubiquitous pathways serve several functions including those involved in energy generation as well as biosynthesis of cofactors, carriers, and vitamins. They also provided capacity for biosynthesis and degradation of nutrient sources such as amines and polyamines, amino acids, carbohydrates, fatty acid and lipids, nucleosides and nucleotides as well as secondary metabolites. Pathways that enabled bacteria to degrade unlikely compounds such as certain alcohols, C1 compounds, carboxylates, inorganic nutrients, and polysaccharides were also shared across these body sites.

A notable finding in this analysis was that body-site-associated, microbial, functional capacity consisted of diverging reaction pathways within shared super classes across the three environments. This was embodied in 10 of the super pathway classes to which some of the shared pathways belonged. These super classes also contributed pathways whose patterns of enrichment showed significant association with body sites. They include pathways of Amine and Polyamine Biosynthesis, Amino Acid Biosynthesis, Carbohydrate Biosynthesis, Carbohydrate Degradation, Cell Structure Biosynthesis, Cofactor, Carrier, and Vitamin Biosynthesis, Fatty Acid and Lipid Biosynthesis, Inorganic Nutrient Metabolism, as well as Secondary Metabolite Degradation. It is typical for many, taxonomically distinct microorganisms within an ecosystem to have the potential to perform similar metabolic functions. This phenomenon is described as ‘functional redundancy’ [49, 50]. However, their pathways and strategies of metabolism are dynamic and can vary with availability of, and quality of resources as well as competition within the community [51]. Therefore, it is not surprising that the distinguishing pathways in these communities, also belong to similar super pathway classes. However, it is also worth noting that presence of a predicted microbial pathway within a community does not necessarily signify its importance in the ecosystem. Unless necessary, many genes/enzymes are not expressed since gene expression is costly and comes as a trade-off with an organism’s fitness [52, 53]. This gap in interpretation can be closed, at least in part, by applying further “omics” techniques. Techniques that probe for proteins/enzymes (proteomics) and metabolites (metabolomics) being produced and therefore, what reactions are occurring within a microbial ecosystem.

## Conclusion

In this work we have presented a comprehensive description of the oropharyngeal, proximal colonic and vaginal microbiome of the Jeju Black pigs. We described both core features within these communities as well as features that are ubiquitous across the studied body sites of these pigs. Among the ubiquitous features, we found members belonging to the genera *Streptococcus*, *Bacillus*, *Lactobacillus*, and *Clostridium* which are commonly used as probiotics. This then, presents an opportunity for concurrently modulating the JBP’s oropharyngeal, colonic, and vaginal microbiomes using in-feed probiotics comprising bacterial strains from these genera. However, our study had a few limitations that should be pointed out. First, we recruited a relatively homogenous group of Korean Native Black pigs. The piglets were born to sows within the same farm and were raised under similar environments including the controlled conditions of a research station. This therefore limits extensibility of these findings to Korean Native pigs raised under varying systems such as free range, deep litter systems among others. Secondly, our comparison with other breeds was only descriptive since the study design did not explicitly compare the microbiota across various pig breeds. Further, it was difficult to compare findings due to differences in design with previously published work. Therefore, our conclusion of what the “core” microbiome of Korean Native Black pigs should be interpreted with these limitations in mind. Nonetheless, our work provides a basis for future studies into the microbiome of this native breed of pigs.

Future studies designed to include pigs raised under defined environments on the Jeju Island and elsewhere in the country are recommended to consolidate our understanding of the microbiome of this breed and facilitate their management. Also, research that takes on a meta-analytical look at the microbiome of the Jeju Black pig with respect to other domestic breeds of pigs will provide a more in-depth understanding of breed as an influencing factor on the porcine microbiome.

## Methods

### Study animals and sampling

The study included nine 7-month-old Jeju Black pig (JBP) gilts that had been held at the Cronex Co. Ltd.’s research facility for 3 months prior to this study. Animal handling and study protocols were conducted in accordance with the Cronex Co., Ltd. Animal Care and Use Committee guidelines. During this period, the animals were housed in a specific pathogen free (SPF) facility with an internal environmental temperature and relative humidity adjusted to 22 ± 2 °C and 50 ± 10%, respectively, under a 12-h light–dark cycle. The animals were allowed ad libitum access to water and a feed based on corn, soybean, and wheat (Table S10). To collect these samples, each of the animals was humanely euthanized by administering an overdose of pentobarbital sodium (100 mg/kg) followed by exsanguination. Swabs were then aseptically taken from the oropharyngeal cavity, and vaginal canal. Proximal colonic contents were collected after dissection. The samples were kept at -20 °C for about a week until DNA extraction was done in the lab.

### DNA extraction and amplification of bacterial 16S rRNA

Total microbial DNA was extracted from the oropharyngeal and vaginal swab samples using the QIAamp® DNA Mini kit (Qiagen, Germantown, MD, USA), following the manufacturer’s instructions. To isolate total genomic DNA from the proximal colonic content, we used the NucleoSpin® DNA Stool Kit (Macherey–Nagel) in accordance with the it’s manual. Purity and quantification of the extracted DNA was then assessed by spectrophotometry using the DropSense™ 96 (Trinean, Gentbrugge, Belgium).

To describe microbial composition, we first targeted and amplified the V3-V4 hypervariable region of the 16 S rRNA gene using polymerase chain reactions (PCR). PCR amplification of this region was achieved using the universal prokaryotic primers Bakt_341F (5′-CCTACGGGNGGCWGCAG-3′), and Bakt_805R (5′-GACTACHVGGGTATCTAATCC-3′) (Primers) [54] containing Illumina overhang adapters. The illumina adapter sequences were 5′-TCGTCGGCAGCGTCAGATGTGTATAAGAGACAG, for the forward primer and 5′-GTCTCGTGGGCTCGGAGATGTGTATAAGAGACAG for the reverse primer. We used 25 μl PCR reaction volumes, each containing 2.5 μl of microbial DNA, 5 μl of each primer at a 1 μM concentration as well as 12.5 μl of 2xKAPA HiFi HotStart ReadyMix. The thermocycler conditions were set at an initial denaturation temperature of 95 °C for 3 min, 25 cycles of 95 °C for 30 s, annealing at 55 °C for 30 s and elongation at 72 °C for 30 s with a final elongation time of 5 min. PCR clean-up was done using the Agencourt AMPure XP beads (Beckman Coulter, Brea, CA, USA) following the manufacturer’s protocol. The PCR products were then kept at ‐20 °C until library preparation and sequencing were done.

### Library preparation and high throughput sequencing

Library preparation and sequencing was conducted at Life is Art of Science (LAS) Laboratory (Gimpo, South Korea). Here, a second PCR reaction that would tag dual indices and illumina sequencing adapters to the amplicons was performed to enable multiplex sequencing. In this PCR stage, a 50 μl reaction volume containing 5 μl of DNA, 5 μl of Nextera XT Index Primer 1 (N7xx), 5 μl of Nextera XT Index Primer 2 (S5xx), 25 μl of 2 × KAPA HiFi HotStart Ready Mix and 10 μl of PCR Grade water. The reaction was run through an initial denaturation step of 3 min at 95 °C followed by 8 cycles of denaturation (95 °C for 30 s), annealing (55 °C for 30 s) and extension (72 °C for 30 s) followed by a final elongation step at 72 °C for 5 min. Another PCR clean up step was performed, using AMPure XP beads (Beckman Coulter, Brea, CA, USA) to clean up the library before quantification and sequencing.

Quality assessment and fragment size validation of the PCR products was accomplished using a microfluidic electrophoresis using the Agilent 2100 Bioanalyzer in combination with the Agilent High sensitivity DNA chips (Agilent Technologies Inc., Santa Clara, CA, USA). DNA purity and concentration were determined using the Fragment Analyzer™ and the dsDNA 910 Reagent Kit, 35 bp—1,500 bp (Advanced Analytical technologies, Inc (AATI), Ames, USA). Equimolar (2 nM) volumes (5 μl) of the indexed and barcoded amplicon libraries from each sample were then pooled and sequenced using the MiSeq v3 reagent kit on an Illumina MiSeq platform (Illumina, San Diego, CA, USA). Sequencing was done using a 2 × 301 bp paired-end sequencing method.

### Bioinformatic analysis

Demultiplexed raw fastq sequence data was preprocessed and analyzed in the open-source pipeline, Quantitative Insights Into Microbial Ecology (QIIME2) using versions, 2021.2 and 2021.11 of the software [55]. The fastq files were quality filtered using dada2 [56] generating amplicon sequence variants (ASV’s) that were used downstream. A phylogenetic tree was constructed using a fragment insertion method based on as SATé-enabled phylogenetic placement (SEPP) technique that is employed in the *q2-fragment-insertion* plugin [57]. The technique builds the tree using a backbone tree from the Greengenes reference database [58]. Taxonomies were assigned using the q2-feature-classifier plugin [59] which is based on the classify-sklearn naïve bayes taxonomy classifier. The classifier was trained on the Greengenes 13_8 99% OTU’s reference sequences trimmed to the V4 hypervariable region of the 16 s rRNA gene [58]. We also included animal secretion-specific taxonomic weights from the *readytowear* repository (https://github.com/BenKaehler/readytowear) which were assembled using *q2-feature-classifier* [59]. Using the environment-specific weights was intended to improve accuracy of the trained classifier by priming it on typical animal secretion sample microbial composition [60]. We then filtered and eliminated all ASVs that had been taxonomically assigned to mitochondria and chloroplasts before further analysis.

Alpha and beta diversity were analyzed using the *diversity* plugin. First, rarefaction curves were plotted to determine an appropriate sampling depth, to which the samples could be normalized as a control for the effects of uneven number of features per sample in diversity calculations. The samples were then normalized to a sampling depth of 12,411feature. To determine the distribution of these samples, we used both the Bray–curtis [61] and the weighted UniFrac distances [62, 63]. With these distance metrics, we constructed principal coordinate analysis plots and visualized them using the Emperor tool [64]. Testing for statistical significance within the observed differences in beta diversity was done using PERMANOVA [65] as implemented in the diversity plugin within QIIME2.

Finally, we used PICRUSt (phylogenetic Investigation of Communities by Reconstruction of Unobserved States) implemented in the q2-picrust2 plugin [66, 67], to predict the functional composition of the microbial communities. KO metagenomes, EC metagenomes and Meta Cyc pathway abundances.

### Statistical analysis

Output from QIIME 2 was imported into R statistical software [68] for further statistical analysis and generation of figures. We mainly used the vegan [69], edgeR in Bioconductor [22], Tidyverse (ggplot2) for analysis in R. Statistical analysis of alpha diversity indices among groups were performed using the Kruskal–Wallis test [70]. Wilcoxon Rank-Sum Tests were later used for paired comparisons between groups [71]. For statistical testing in beta diversity, we used the Adonis method [65] implemented in the vegan package. To correct for multiple hypothesis testing we used the Benjamini–Hochberg False Discovery Rate (FDR) method at a cutoff of 0.05 for statistical significance, unless otherwise noted.

## Supplementary Information

**Additional file 1:** **Table S1.** Denoising statistics of the sequences that passed the quality filtering process.

**Additional file 2: Table S2.** Genera that are significantly associated with the oropharyngeal cavity. **Table S3.** Genera that are significantly associated with the proximal colon. **Table S4.** Genera that are significantly associated with the vaginal canal.

**Additional file 3: Table S5.** The most abundant microbial pathways within the oropharyngeal cavity, proximal colon, and vaginal canal of 7-month-old Jeju Black Pig gilts.

**Additional file 4: Table S6.** Pathways that are significantly associated with the oropharyngeal cavity. **Table S7.** Pathways that are significantly associated with the proximal colon. **Table S8.** Pathways that are significantly associated with the vaginal canal.

**Additional file 5: Table S9.** Shared MetaCyc microbial pathways within the oropharyngeal cavity, proximal colon, and vaginal canal of 7-month-old Jeju Black Pig gilts.

**Additional file 6: Table S10.** Composition, nutrient, and energy contents of feed ration availed to the study animals. **Figure S1.** Shared amplicon sequence variants (ASV’s) across body sites of the Jeju Black Pig gilts.

**Additional file 7: Table S11. **Studies on the porcine oropharyngeal microbiome. **Table S12.** Studies on the porcine colon microbiome. **Table S13.** Studies on the porcine vaginal microbiome.

## Data Availability

Raw sequences are available from Sequence Read Archive (SRA) with the Bioproject accession number PRJNA827006 (https://www.ncbi.nlm.nih.gov/sra/PRJNA827006).
