# Supplementary material for: Oropharyngeal, proximal colonic, and vaginal microbiomes of healthy Korean native black pig gilts
Source: BMC Microbiol. 2023 Jan 5;23:3. doi: 10.1186/s12866-022-02743-3 (PMC9814203; doi:10.1186/s12866-022-02743-3)
Supplement: Supplementary file 6 — Additional file 6: Table S10. Composition, nutrient, and energy contents of feed ration availed to the study animals. Figure S1. Shared amplicon sequence variants (ASV’s) across body sites of the Jeju Black Pig gilts. [file 12866_2022_2743_MOESM6_ESM.docx]

**Additional Information 6**

**Oropharyngeal, Proximal colonic, and Vaginal Microbiomes of Healthy Korean Native Black Pig Gilts**

**Andrew Wange Bugenyi^1,2^, Ma-Ro Lee^3^, Yeon-Jae Choi^4^, Ki-Duk Song^5^, Hak-Kyo Lee^3,5^, Young-Ok Son^6,8^, Dong-Sun Lee^7,8^, Sang-Chul Lee^9^, Young-June Son^9^ & Jaeyoung Heo^3^***

^1^Department of Agricultural Convergence Technology, Jeonbuk National University, Jeonju, 54896, Republic of Korea

^2^National Agricultural Research Organization, Mbarara, Uganda

^3^Department of Animal Biotechnology, Jeonbuk National University, Jeonju, 54896, Republic of Korea

^4^International Agricultural Development and Cooperation Center, Jeonbuk National University, Jeonju, 54896, Korea

^5^The Animal Molecular Genetics and Breeding Center, Jeonbuk National University, Jeonju, 54896, Republic of Korea

^6^Department of Animal Biotechnology, Faculty of Biotechnology, College of Applied Life Sciences and Interdisciplinary Graduate Program in Advanced Convergence Technology and Science, Jeju National University, Jeju, 63243, Republic of Korea

^7^Faculty of Biotechnology, College of Applied Life Sciences and Interdisciplinary Graduate Program in Advanced Convergence Technology and Science, Jeju National University, Jeju, 63243, Republic of Korea

^8^Jeju Microbiome Research Center, Jeju National University, Jeju Special Self-Governing Province, 63243, Republic of Korea

^9^Cronex Co., Cheongju, 28174, Republic of Korea **Feeding of study animals**

**Table S10; Composition, nutrient, and energy contents of feed ration availed to the study animals**

| **Nutrient** | **Basal Diet** |
| --- | --- |
| **Ingredients, g/kg** |  |
| Corn | 392.5 |
| Wheat | 200.0 |
| Soybean meal | 137.3 |
| Wheat bran | 104.0 |
| Soybean hull | 54.1 |
| Rice bran | 40.0 |
| Fiber feed | 20.0 |
| Limestone | 15.6 |
| Tallow | 10.0 |
| Mono-dicalcium phosphate | 9.0 |
| NaCl | 5.0 |
| Choline chloride | 2.2 |
| Acidifier | 2.0 |
| Tryptophan | 2.0 |
| Lysin | 1.8 |
| Toxin-binder | 0.5 |
| Vitamin Premix ^a^ | 2.0 |
| Mineral Premix ^b^ | 2.0 |
| Total | 1,000.00 |
| **Analyzed crude nutrient and energy contents** |  |
| Moisture, % | 12.98 |
| Crude protein, % | 15.65 |
| Crude fat, % | 3.58 |
| Crude ash, % | 2.83 |
| Crude fiber, % | 5.64 |
| Gross energy, Kcal/kg | 3,836.00 |

**^a^** The vitamin premix provided per kg diet: vitamin A, 12,000 IU; vitamin D3, 2,000 IU; vitamin E, 100IU; vitamin K3, 4.5mg; vitamin B1, 2mg; vitamin B2, 7mg; vitamin B3, 45mg; pantothenic acid, 30mg; vitamin B6, 4.5 mg; Biotin, 0.5mg; Folic acid, 3.5 mg; vitamin B12, 0.03 mg; antioxidant, 6.6mg

**^b^** The mineral premix provided per kg diet: Fe, 150mg; Zn, 85mg; Mn, 37mg; Cu, 11mg; Co, 2mg; I, 0.3mg; Se, 0.15mg

## **Additional discussion on ubiquitous features.**


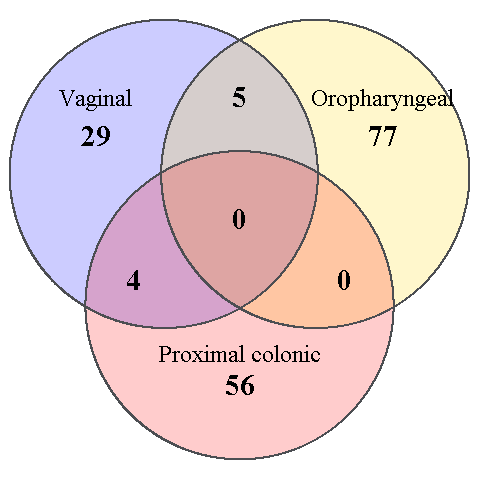


**Figure 1: Shared amplicon sequence variants (ASV’s)** across body sites of the Jeju Black Pig gilts.

The core microbiota in the oropharyngeal, proximal colonic, and vaginal communities was composed of a total of 82, 60, and 38 ASV’s, respectively. These corresponded to 25, 21, and 17 genera, respectively. Unlike operational taxonomic units (OUT) that are usually within a similarity of 97%, the DADA2 algorithm employed in QIIME2 infers exact amplicon sequence variants (ASV) with the ability to resolve biological differences in sequences to within variations of 1 or 2 nucleotides [1, 2]. However, this level of resolution may not necessarily be advantageous as the classification of bacteria using 16SrRNA data becomes less reliable below the genus level [3]. Besides, the definition of a species in Bacteria is still more or less ambiguous [4-6]. With these considerations, we chose to restrict our interpretation of results to the genus level.

## **References**

1. Callahan, B.J., P.J. McMurdie, and S.P.J.T.I.j. Holmes, *Exact sequence variants should replace operational taxonomic units in marker-gene data analysis.* 2017. **11**(12): p. 2639-2643.

2. Callahan, B.J., et al., *DADA2: High-resolution sample inference from Illumina amplicon data.* 2016. **13**(7): p. 581-583.

3. Schloss, P.D.J.M., *Amplicon sequence variants artificially split bacterial genomes into separate clusters.* 2021. **6**(4): p. e00191-21.

4. Bapteste, E., et al., *Prokaryotic evolution and the tree of life are two different things.* 2009. **4**(1): p. 1-20.

5. Bobay, L.-M.J.T.P., *The prokaryotic species concept and challenges.* 2020: p. 21-49.

6. Doolittle, W.F. and O.J.G.r. Zhaxybayeva, *On the origin of prokaryotic species.* 2009. **19**(5): p. 744-756.
