# Supplementary material for: Oropharyngeal, proximal colonic, and vaginal microbiomes of healthy Korean native black pig gilts
Source: BMC Microbiol. 2023 Jan 5;23:3. doi: 10.1186/s12866-022-02743-3 (PMC9814203; doi:10.1186/s12866-022-02743-3)
Supplement: Supplementary file 7 — Additional file 7: Table S11. Studies on the porcine oropharyngeal microbiome. Table S12. Studies on the porcine colon microbiome. Table S13. Studies on the porcine vaginal microbiome. [file 12866_2022_2743_MOESM7_ESM.docx]

**Additional Information 7**

**Oropharyngeal, Proximal colonic, and Vaginal Microbiomes of Healthy Korean Native Black Pig Gilts**

**Andrew Wange Bugenyi^1,2^, Ma-Ro Lee^3^, Yeon-Jae Choi^4^, Ki-Duk Song^5^, Hak-Kyo Lee^3,5^, Young-Ok Son^6,8^, Dong-Sun Lee^7,8^, Sang-Chul Lee^9^, Young-June Son^9^ & Jaeyoung Heo^3^***

^1^Department of Agricultural Convergence Technology, Jeonbuk National University, Jeonju, 54896, Republic of Korea

^2^National Agricultural Research Organization, Mbarara, Uganda

^3^Department of Animal Biotechnology, Jeonbuk National University, Jeonju, 54896, Republic of Korea

^4^International Agricultural Development and Cooperation Center, Jeonbuk National University, Jeonju, 54896, Korea

^5^The Animal Molecular Genetics and Breeding Center, Jeonbuk National University, Jeonju, 54896, Republic of Korea

^6^Department of Animal Biotechnology, Faculty of Biotechnology, College of Applied Life Sciences and Interdisciplinary Graduate Program in Advanced Convergence Technology and Science, Jeju National University, Jeju, 63243, Republic of Korea

^7^Faculty of Biotechnology, College of Applied Life Sciences and Interdisciplinary Graduate Program in Advanced Convergence Technology and Science, Jeju National University, Jeju, 63243, Republic of Korea

^8^Jeju Microbiome Research Center, Jeju National University, Jeju Special Self-Governing Province, 63243, Republic of Korea

^9^Cronex Co., Cheongju, 28174, Republic of Korea

**Table S11:** **Studies on the porcine oropharyngeal microbiome**

| **Reference** | **No. of animals** | **Breed** | **Age at sampling** | **Body site(s)** | **Sample type(s)** | **Hypervariable region** | **Sequencing platform** | **Data processing software** | **Reference database** | **Main taxonomic composition (Phylum level)** | **Main taxonomic composition (Genus level)** |
| --- | --- | --- | --- | --- | --- | --- | --- | --- | --- | --- | --- |
| [1] | 8 | [Hampshire x Yorkshire], Purebred Yorkshire, & [Hampshire x Camborough] | 18 - 20 weeks | Oropharyngeal cavity | tonsil tissues & swabs | full length | Roche GS-FLX system | RDP pyrosequencing pipeline | RDP | *Proteobacteria* (73.4%),  *Firmicutes* (17.8%),  *Fusobacteria* (5.6%) | *Actinobacillus* (36.99%), *Pasteurella* (16.06%), *Alkanindiges* (12.00%), *Fusobacterium* (6.96%), *Haemophilus* (6.62%), *Lactobacillus* (3.61%), *Veillonella* (3.17%), *Peptostreptococcus* (2.58%), *Moraxella* (0.81%), *Streptococcus* (0.63%) |
| [2] | 8 | Not specified | Slaughtered pigs | Cervical lymphatic tissue | Palatine tonsil residues & mandibular lymph node biopsies | V1-V2 | GS-FLX Titanium | Mothur | SILVA | ***Abundant phyla in the tonsils;*** *Bacteroidetes* (36.58%), *Proteobacteria* (33.09%),  *Firmicutes* (14.27%) | ***Abundant genera in the tonsils;*** *Prevotella* (17.65 ± 5.27), *Porphyromonas* (12.55 ± 3.36), *Campylobacter* (12.12 ± 4.40)*, Treponema* (11.49 ± 4.25), *Streptococcus* (8.04 ± 3.87), *Serratia* (6.57 ± 3.49), *Paraprevotella* (4.97 ± 1.35), *Bacteroides* (4.79 ± 1.57), *Fusobacterium* (3.92 ± 1.00) |
| [3] | 33 | [Landrace × Yorkshire × Duroc] & [Landrace × Yorkshire × Hampshire] | 2-3 weeks | Oropharyngeal cavity | Oropharyngeal swabs | V3-V4 | Illumina MiSeq platform | Mothur | SILVA | *Firmicutes* (53.11%),  *Proteobacteria* (27.89%),  *Bacteroidetes* (12.17%),  *Fusobacteria* (3.15%),  *Actinobacteria* (2.29%) | *Lactobacillus (*30.44 - 37.28%*),  Streptococcus* (23.60 - 32.26%)*,  Actinobacillus* (11.48 - 15.09%) |
| [4] | 8 | [Landrace × Yorkshire × Duroc] | 1-7 weeks | Oropharyngeal cavity | Oropharyngeal swabs | V4 | Illumina MiSeq platform | QIIME | Greengenes | *Proteobacteria* (52.59%), *Firmicutes* (33.59%),  *Bacteroidetes* (7.32%),  *Actinobacteria* (2.20%) | *Streptococcus* (17.38%), *Moraxella* (13.00%), *Haemophilus* (8.97%), *Actinobacillus* (7.52%), *Veillonella* (2.62%) |

**Table S12: Studies on the porcine colon microbiome**

| **Reference** | **No. of animals** | **Breed** | **Age at sampling** | **Body site(s)** | **Sample type(s)** | **Hypervariable region** | **Sequencing platform** | **Data processing software** | **Reference database** | **Main taxonomic composition (Phylum level)** | **Main taxonomic composition (Genus level)** |
| --- | --- | --- | --- | --- | --- | --- | --- | --- | --- | --- | --- |
| [5] | 24 | Duroc × Landrace × Large White | Not specified | Ileum, cecum & colon | Mucosa & luminal contents of distal ileum, cecum & proximate colon | V3–V4 | Illumina MiSeq PE300 platform | Mothur | RDP | ***In the colon luminal content;*** *Firmicutes* (53.48±2.38%) *Proteobacteria* (4.1±0.42%) *Bacteroidetes* (40.09±2.32%) | ***In the colon luminal content;*** *Prevotella_9 (14.98%), Megasphaera (7.84%), Alloprevotella (6.13%), LactobacillPrevotella_9 (14.98%), Megasphaera (7.84%), Alloprevotella (6.13%), Lactobacillus (5.27%), Prevotellaceae_NK3B31_group (4.03%), Pseudobutyrivibrio (3.93%), Anaerovibrio (3.37%), Prevotellaceae_uncultured (2.5%), Faecalibacterium (2.33%), Bacteroidales_S24-7_group_norank (2.3%)* |
| [6] | 16 | Large White &  Ningxiang | 20 weeks | Colon | Colonic content | V3-V4 | Illumina MiSeq 2500 platform | QIIME | SILVA | *Firmicutes* (47.36–74.27%)  *Bacteroidetes* (20.41–36.64%),  *Spirochaetes* (0.75–27.11%), *Proteobacteria* (0.87–1.94%) | *Lactobacillus,  Clostridium,  Terrisporobacter  Treponema* |
| [7] | 12 | Tibetan & Yorkshire | Adults | Colon | Colonic content | V3–V4 | NovaSeq 6000 | RDP classifier | SILVA | *Firmicutes* (81.15 & 76.26%),  *Bacteroidetes* (6.80 & 6.44%) ***in Tibetan & Yorkshire, respectively.*** | *Clostridium sensu stricto 1* (21.08% & 7.73%),  *Lactobacillus* (10.44% & 1.83%), *Sporobacillus* (6.98% & 4.53%), *Streptococcus* (6.50% & 2.90%)*,  Ruminococcaceae_UCG-005* (3.36% & 2.36%) ***in Tibetan & Yorkshire, respectively.*** |

**Table S13: Studies on the porcine vaginal microbiome**

| **Reference** | **No. of animals** | **Breed** | **Age at sampling** | **Body site(s)** | **Sample type(s)** | **Hypervariable region** | **Sequencing platform** | **Data processing software** | **Reference database** | **Main taxonomic composition (Phylum level)** | **Main taxonomic composition (Genus level)** |
| --- | --- | --- | --- | --- | --- | --- | --- | --- | --- | --- | --- |
| [8] | 20 | Göttingen Minipigs | 12 weeks (prepubertal) 52 weeks (sexually mature) | Vaginal tract | Vaginal swabs | V3–V4 | Illumina MiSeq platform | MG-RAST | M5NR database | ***In prepubertal:*** *Firmicutes* (49.3%),  *Proteobacteria* (35.2%),  *Tenericutes* (6.3%),  *Actinobacteria* (5.7%) &  *Bacteroidetes* (3.5%) ***In sexually mature:*** *Firmicutes* 48.1% (42.8–52.7%),  *Proteobacteria* 37.7% (32–40.3%),  *Actinobacteria* 7.6% (5.9–10.7%),  *Bacteroides* 5.4% (4.2–6.8%) &  *Tenericutes* 1.3% (0.6–2%) | ***In prepubertal:*** Unclassified *Gammaproteobacteria* (8.5%),  *Paenibacillus* (8.4%),  *Mesoplasma* (6.3%),  *Proteus* (6.3%),  *Lactobacillus* (6%),  *Listeria* (5.7%) ***In sexually mature:*** Unclassified *Gammaproteobacteria* 10.10% (9.6 - 10.6%), Unclassified *Clostridiales Family XI. Incertae Sedis* 9.05% (8.2 - 10.5%),  *Yersinia* 8.72% (5.5 - 12.3%),  *Paenibacillus* 6.60% (5.1 - 7.2%), *Syntrophus* 6.68% (5.9 - 8.6%), *Heliobacterium* 6.75% (4.9 - 9.3%),  *Listeria* 6.23% (5.7 - 7.7%),  *Faecalibacterium* 6.25% (4.1 - 7.4%),  *Proteus* 5.5% (4.0 - 6.8%) |
| [9] | 8 | Landrace | Third parity sows | Vaginal tract | Vaginal swabs | V3 | Illumina MiSeq platform | RDP Classifier, QIIME | RDP | *Firmicutes* (60.88% & 45.86%)  *Proteobacteria* (20.45% & 32.19%)  *Bacteroidetes* (9.19% & 12.99%)  in healthy & endometritis sows, respectively | *Bacillus,  Paenibacillus,  Alkaliphilus,  Cronobacter*   in healthy sows |
| [10] | 50 | Landrace × Yorkshire | 2nd -7th parity sows | Vaginal tract | Vaginal swabs | V4 | Illumina MiSeq platform | RDP Classifier, Mothur | RDP, Greengenes | *Firmicutes* (44.51%),  *Proteobacteria* (33.68%),  *Bacteroidetes* (9.26%) | *Psychrobacter* (12.22%),  *Escherichia* (5.92%),  *Pseudomonas* (5.82%),  *SMB53* (5.72%), *Anaerococcus* (4.03%) |
| [11] | 27 | Duroc × [Landrace × Yorkshire] | gilts & pregnant sows | Vaginal tract | Vaginal swabs | V3-V4 | Illumina MiSeq platform | QIIME | Greengenes | *Proteobacteria* (33.1%), *Firmicutes* (32%),  *Cyanobacteria* (13.3%),  *Actinobacteria* (13.2%),  *Fusobacteria* (1.9%),  *Chloroflexi* (1.6%),  *Bacteroidetes* (1.5%) & *Gemmatimonadetes* (1.1%) | *Lactobacillus,  Acinetobacter,  Burkholderia,  Fusobacterium,  Clostridium,  Bacillus,  Streptococcus,  Enterobacter,  Pseudomonas,  Enterococcus,  Sphingomonas, Averyella* |
| [12] | 20 | Not specified | Sows | Vaginal tract | Vaginal swabs | V3-V4 | Illumina MiSeq platform | RDP Classifier | SILVA | *Firmicutes* (40.76%),  *Proteobacteria* (34.21%),  *Bacteroidetes* (11.23%),  *Actinobacteria* (7.62%),  *Fusobacteria* (1.89%) | *Anaerococcus* (1.91 - 10.52%), *Terrisporobacter* (0.74 - 4.49%), *Jeotgalicoccus* (0.08 - 3.71%), *Enterococcus* (0.01 - 4.41%), *Finegoldia* (0.29 - 8.03%), *Peptoniphilus* (0.41 - 6.82%), *Ezakiella* (0.40 - 4.36%), *Streptococcus* (2.07 - 6.97%), *Clostridium_sensu_stricto_1* (1.21 - 6.72%), *Staphylococcus* (0.15 - 4.07%), *Actinobacillus* (0.2 - 6.91%), *Campylobacter* (0.40 - 8.46%), *Bacteroides* (1.08 - 6.24%), *Porphyromonas* (1.10 - 10.98%), *Fusobacterium* (0.78 - 3.57%), *Corynebacterium_1* (1.23 - 3.67%) |

References

1. Lowe, B.A., et al., *Defining the" core microbiome" of the microbial communities in the tonsils of healthy pigs.* 2012. **12**(1): p. 1-14.

2. Mann, E., et al., *The metabolically active bacterial microbiome of tonsils and mandibular lymph nodes of slaughter pigs.* 2015. **6**: p. 1362.

3. Wang, Q., et al., *Comparison of oropharyngeal microbiota in healthy piglets and piglets with respiratory disease.* 2018. **9**: p. 3218.

4. Bugenyi, A.W., et al., *Association between oropharyngeal microbiome and weight gain in piglets during pre and post weaning life.* 2020. **62**(2): p. 247.

5. Zhang, L., et al., *Spatial heterogeneity and co-occurrence of mucosal and luminal microbiome across swine intestinal tract.* 2018. **9**: p. 48.

6. Lei, L., et al., *Comparative microbial profiles of colonic digesta between ningxiang pig and large white pig.* 2021. **11**(7): p. 1862.

7. Shang, P., et al., *Healthy Gut Microbiome Composition Enhances Disease Resistance and Fat Deposition in Tibetan Pigs.* 2022. **13**.

8. Lorenzen, E., et al., *The vaginal microbiome is stable in prepubertal and sexually mature Ellegaard Göttingen Minipigs throughout an estrous cycle.* 2015. **46**(1): p. 1-13.

9. Wang, J., et al., *Characterization of vaginal microbiota of endometritis and healthy sows using high-throughput pyrosequencing of 16S rRNA gene.* 2017. **111**: p. 325-330.

10. Zhang, J., et al., *Gut and Vagina Microbiota Associated With Estrus Return of Weaning Sows and Its Correlation With the Changes in Serum Metabolites.* 2021. **12**.

11. Luque, A.T., et al., *Vaginal bacterial diversity from healthy gilts and pregnant sows subjected to natural mating or artificial insemination.* 2021. **140**: p. 26-37.

12. Liang, H., et al., *High-throughput sequencing of 16S rRNA gene analysis reveals novel taxonomic diversity among vaginal microbiota in healthy and affected sows with endometritis.* 2022. **143**: p. 33-40.
